# Rights and responsibilities: Women leadership for health in Kyrgyzstan

**DOI:** 10.1371/journal.pone.0295239

**Published:** 2024-02-16

**Authors:** Vesna Bjegovic-Mikanovic, Sanja Matovic-Miljanovic, Chinara Seitalieva, Tatyana Makarova, Gulgun Murzalieva, Kanatbek Kozhokeev, Helmut Wenzel, Ulrich Laaser

**Affiliations:** 1 Faculty of Medicine, University of Belgrade, Belgrade, Serbia; 2 Euro Health Group, Denmark / Regional Office Belgrade, Serbia; 3 SDC Funded Project‚ Health Facilities Autonomy Project Phase II [exit phase], Kyrgyzstan, Germany; 4 Independent consultant, Konstanz, Germany; 5 Faculty of Health Sciences, University of Bielefeld, Bielefeld, Germany; Torrens University Australia, NEPAL

## Abstract

The World Health Organization (WHO) is committed to empowering countries by implementing a gender, equity, and human rights approach in the health sector. The objective of this gender and inclusion analysis is to assess potential gender disparities of health sector management in the Kyrgyz Republic. The employed mixed-method approach takes advantage of data triangulation. Besides information from the literature and policy documents available at the international and national levels, the analysis includes interviews and data from the self-assessment of health services managers in the Kyrgyz Republic. A convenience sample of 75 health managers was taken and after up to three reminders a commendable response rate of 80% was achieved which resulted the final sample size of N = 60. A factor analysis using quartimax orthogonal rotation was applied to investigate the correlation between Teaching Qualification, Digitalization, Training Usefulness, Computer Workplace, and Gender Equality. In 2021, the Kyrgyz Republic adopted a new Constitution, which provides a sound legal framework to support gender equality and promote women’s empowerment. However, according to a survey, only 42.9% of the respondents felt that equal rights and opportunities were integrated into their job descriptions. Similarly, only 40.7% believed that their institutions’ written documents reflected a commitment to equal rights and opportunities for both genders. Two factors were identified as influencing gender equality: (1) personal and (2) technical aspects. Regarding personal aspects, gender equality, teaching qualification, and training usefulness were found to be significant. Regarding technical aspects, the computer workplace was related. In recent years, the Kyrgyz Republic has been developing a culture of gender equality. Political will is essential to promote and make organizational change possible. It is important to create a written mid-term policy that affirms a commitment to gender equality in organizational behavior, structures, staff, and management board compositions. Healthcare institutions need to prepare strategic and operational plans that incorporate gender equality principles.

## Introduction

Many countries take inspiration from the UN agenda for sustainable development [1], especially from Sustainable Development Goal (SDG) 3 on health and well-being and SDG 5, dedicated to gender equality. Modern strategies for health system development try to empower women and promote gender equality in governance and management at the macro (society and policy), meso (communities and institutions), and micro (social interaction in departments) levels [2]. The World Health Organization’s (WHO) approach to gender mainstreaming [3] refers to projects and institutions striving to build capacities in developing gender equality, promoting the use of sex-disaggregated data and gender analysis, and establishing accountability. Since women account, on average, for 70% of the health workforce [4], gaps in health workers will decrease only by addressing the gender dynamics of the workforce. In its 5-year Strategy (2019–2023), WHO [5] is committed to empowering countries for gender equity and a human rights approach in the health sector’s day-to-day activities. A recent review [6] pointed out that female health workers who deliver most of the care in all settings face barriers that their male colleagues do not. This situation not only undermines their well-being and livelihood but also constrains progress on gender equality and negatively impacts health systems and the delivery of quality health services. Gender parity in health management differs from country to country, but men always prevail in the top health services positions [7]. Similar disparities between females and males apply to work reimbursement; women are usually underpaid [8]. We use the term "Responsibilities" here to refer to the level of positions held by women.

To speed up interventions for gender equality, in 2017, WHO established the Gender Equity Hub (GEH), co-chaired by WHO and Women in Global Health under the Global Health Workforce Network umbrella. The GEH brings together key stakeholders to strengthen gender-transformative policy guidance and implementation capacity for overcoming gender biases and inequalities in the health workforce—in support of implementing the Global Strategy on Human Resources for Health: Workforce 2030 [5]. Gender analysis, empowerment, and mainstreaming became significant cross-cutting issues in developing capacity for health system management. Much evidence confirms that a lack of gender parity in higher-level decision-making positions and leadership [9] can influence the efficiency and quality of health services. In contrast, discrimination in health service settings can compromise Universal Health Coverage [6].

Still, gender inequalities hinder progress and influence women’s leadership in many countries. Gender inequalities in management refer to the unequal treatment or discrimination of individuals based on gender within a management or leadership role [10]. They include unequal pay, limited opportunities for promotion or advancement, and biased evaluations or performance appraisals. The representation of women is not proportional to the overall demographics of the health workforce. Gender inequality in management can negatively impact individual employees and the organization, leading to lower morale, decreased productivity, and a lack of diversity in decision-making processes. In the Kyrgyz Republic, like in many countries, there is a significant gender imbalance in leadership positions in the health sector. Women comprise most of the health workforce but are often underrepresented in management and leadership roles. This situation can lead to gender inequalities in terms of pay, opportunities for advancement, and decision-making power. The specific context of Kyrgyzstan can influence gender equality in management in several ways. One factor is the country’s cultural and social context, which contributes to traditional gender roles and expectations that can impact women’s opportunities for leadership and advancement. Another factor is the country’s economic context. Kyrgyzstan has a relatively low-income economy, and this can limit opportunities for women and make it more difficult for them to access education and training that could help them qualify for management positions.

Additionally, Kyrgyzstan has a history of political instability and conflict, which can create challenges for women seeking to advance in their careers. This aspect includes a lack of access to networking and mentorship opportunities and a lack of support and resources for women-led businesses. While progress has been made in promoting gender equality in Kyrgyzstan, challenges remain. Still, work is to be done until women have equal chances to succeed in management and leadership positions.

The objective of this analysis is to assess the degree of gender disparities at all levels in the health sector management of the Kyrgyz Republic, which can influence efficient health services management, quality, and delivery nota bene: full implementation of the gender-related legislation of the Kyrgyz Republic, equal chances of women and men in competition and appointment, equal remuneration, and job security for women. We look at the policy level, making use of the available governmental documentation and the institutional level through the lenses of stakeholders and directors of health institutions to determine gender equality in governance and management at the macro (society and policy) meso (communities and institutions), and micro (social interaction in departments) levels.

## Methods

### Study design and sources of data

In this section, we outline the study design and diverse sources of data harnessed to provide a foundation for our research. The mix-method takes advantage of data triangulation. The study commenced with a meticulous analysis of gender indicators in the Kyrgyz Republic through a Policy Brief Analysis [11]. Subsequently, an extensive exploration of data sources was undertaken, encompassing inputs from the Ministry of Labor, the Ministry of Health, the National Statistical Committee of the Kyrgyz Republic, and the Institute for Statistical Studies and Continuing Education as well as the United Nations Statistical Database (UNSD). Notably, the UNSD supplied essential data pertaining to SDG 5.5.1 and 5.5.2 targets [12].

Augmenting the existing literature and policy documents, the study gleaned valuable insights from the self-assessment of Ministry of Health [MoH] stakeholders and managers of health services in the Kyrgyz Republic. Information was obtained through a web-based questionnaire from a convenience sample [11] of 75 health managers from a pool of 354 health facilities based on 2019 data from the E-Health Center of the Kyrgyz Republic [13]. Ethical approval by the respective committee was not required, as questionnaires were collected after written informed consent was signed. A commendable response rate of 80% was achieved after up to three reminders which resulted the final sample size of N = 60.

### Research instruments and variables

The questionnaire for health services managers is designed following the UN and WHO approach [1,3], international literature, and instructions related to gender analysis with a focus on occupational segregation, decent work free from bias, discrimination, and harassment, including sexual harassment; gender pay gap; and gender parity in leadership [6,10]. Gender analysis in the Kyrgyz Republic also relates to the complementarities of recommendations in national documents with an international approach. The questionnaire comprised 35 questions divided into five groups: Gender Equality– 13 questions, Teaching Qualification– 3 questions, Digitalization– 4 questions, Training Usefulness –4 questions, and Computer Workplace– 3 questions, and was constructed according to the InterAction Handbook [14]. Besides, eight questions related to personal and professional background. We did expert validation [15] of the questionnaire in which the Kyrgyz professionals’ group, with specialized knowledge and expertise in the health management and gender approach, assessed and evaluated its content, structure, and overall quality. They reviewed content validity, clarity and readability, structure and format, consistency in terminology, and potential biases or culturally insensitive elements that might influence respondents’ answers.

Variables related to Gender Equality were the following: problems of inequality between men and women in selection and appointment to leadership positions, problems of inequality between men and women in earnings in similar leadership positions, other problems of inequality, support to implementing the Law on State Guarantees of Equal Rights and Equal Opportunities for Men and Women by higher authorities, reporting of data differentiated by gender, the inclusion of the equal rights and opportunities in organizational plans and programs, commitment to equal rights in written organizational documents, status of women involvement in leadership positions, existence of the job knowledge, skills and attitudes in gender-sensitive manner, acting in compliance with the state guarantees of equal rights, presence of obligation to the same acting in the job descriptions, serious consideration of equal right by the organizational staff, and compliance with the national regulations in the field of gender policy.

### Statistical analysis

The methodology adopted for calculating the projected achievement of SDG 5.5.1. and 5.5.2 targets by 2030 is detailed in Annex A. SDG 5.5, target relates to “ensuring women’s full and effective participation and equal opportunities for leadership at all levels of decision-making in political, economic, and public life”. Quantifiable indicators for this target were in focus, while in the calculation of gaps in years for achievement, 2010 and 2015 were baseline years, 2020 was the observed year, and 2030 was the targeted year.

All specific variables from the self-assessment related to Gender Equality, Teaching Qualification, Digitalization, Training Usefulness, and Computer Workplace were categorical, provided by their absolute numbers and percentages. We used Chi-square to test the differences between categories. Variables were summed up to present Gender Equality, Teaching Qualification, Digitalization, Training Usefulness, and Computer Workplace. To analyze possible relationships between these five sectors, we employed factor analysis to identify latent structures of the variables and uncover possible hidden connections. We choose the principal component option, which seeks to explain a large part of the total variance of the variables [16]. To improve the interpretation of the factor solutions a quartimax orthogonal rotation was used. Quartimax rotation of variable points aims at improving the factor solutions’ interpretability [16,17]. A successful rotation minimizes the number of factors needed to explain a variable. The software used for data analysis was Statistica, Version 13. TIBCO Software Inc.; 2017 [17].

## Results

The health system leadership in Kyrgyzstan takes responsibility for implementing existing gender equality policies.

### Legislation to promote gender equality

Already in 2003, the Kyrgyz Republic adopted a new Constitution committed to international treaties and agreements. The Constitution was updated in 2022 and included paragraph 24/3 stating that men and women have equal rights, freedoms, and opportunities for their realization. This sound legal framework supports gender equality and promotes women’s empowerment, with a grounded gender approach in the Constitutional Amendments of 2007, 2008, and 2010 [18]. Since then, several laws have been adopted that relate directly to gender equality and women’s empowerment, with the latest Law on State Guarantees of Equal Rights and Equal Opportunities for Men and Women in 2008 establishing State guarantees to ensure gender equality [19]. Furthermore, the Program of the Kyrgyz Republic Government on Public Health Protection and Health Care System Development for 2019–2030 ("Healthy Person–Prosperous Country") [20] is essential in opening a solid base for gender mainstreaming in the health sector. In this framework, assessments of gender policies exist in the Kyrgyz Republic with an obligation for reporting. These laws are the most significant, comprehensive legislation to ensure equality between women and men.

#### The Kyrgyz National Gender Strategy

The Kyrgyz Republic’s first long-term National Gender Strategy (NGS) on Achieving Gender Equality (by 2020) was adopted in 2012 [21]. This Strategy outlined five priorities for sustainable development of gender equality: 1) robust, effective institutional mechanisms; 2) economic empowerment; 3) an education system that promotes gender equality; 4) access to justice for women; and 5) gender-equitable political participation. The new National Sustainable Development Strategy (NSDS) (2018–2040) establishes key objectives to eliminate all disparities, including gender inequality. Cited from the UNDP report of 2018 [18]: "The Kyrgyz Republic has ratified major international conventions on women’s rights and gender equality and occupies a leading position in the Commonwealth of Independent States (CIS) in developing a national legal framework on women’s rights in compliance with international standards".

### Future improvements of gender mainstreaming

Despite progress in establishing a legal and policy framework, a significant space remains to improve gender mainstreaming at the national and institutional levels. According to international data, inequalities continue to be pervasive. Patriarchal attitudes are still prominent; gender inequalities are present in all spheres of social and economic life. The last World Economic Forum Global Gender Gap Report 2020 [22] estimates the Kyrgyz Republic to be in the 93^rd^ position out of 153 countries. Regarding economic participation and opportunities for women, the Kyrgyz Republic is ranked 88^th^, educational attainment 82^nd^, health, survival, and political empowerment 105^th^.

The Kyrgyz Republic Country Gender Assessment in 2019 [23] pointed out that the country has achieved near gender parity in education enrollment and literacy rates, but not regarding the indicators of women’s labor force participation and women’s leadership, i.e., their responsibility level economic participation for women is low at 48.2% compared to 75.7% for men. Women’s participation in political life is also low, with only 16% of seats in the Parliament in 2018. It is worth noting that the representative of the Kyrgyz Republic was one of 28 members of the UN expert group for the formulation of SDG indicators, including those related to gender [24]. Fundamental gender equality issues and challenges, based on the UNDP report [18], are gender stereotypes and socio-cultural norms, women’s economic empowerment, and the gender dimension of human development (here: violence against women, women’s unpaid work and poverty, women resilience and women’s political presentation). Except for Kyrgyz women’s health status and utilization of health services, this report does not highlight the gender situation in health facilities. However, the report indicated that health and social services employees are predominantly female. With over 83.2% employed women, this sector represents the most significant job market for women in the Kyrgyz Republic [25]. In addition, a woman’s leadership development path is often longer than that of men, which is associated with women’s childcare and household management responsibilities. If men can become a leader on average after 8 years, women need 15–18 years, waiting for children to grow up. It should also be noted that women mainly work as deputy heads and heads of primary healthcare organizations, while men lead the secondary and tertiary sectors. No systematic measures have been taken to encourage the promotion of more women in leadership positions.

Based on the national legislation, there should not be any differences between genders regarding the legal conditions of work. Mothers with children under one year can request benefits in terms of working hours associated with feeding a child, and their working day can be reduced by one hour. However, no infrastructural conditions favor breastfeeding by women who work in the health sector although national legislation provides the possibility of parental leave for the father. When planning activities, managers consider the gender of employees in the division of labor, assigning night shifts and job assignments with biological vulnerability, preferably to males, especially in primary health care facilities.

Regarding the possibility of capacity building, women in the Kyrgyz Republic can approach online learning to save time and money and avoid separation from their families. However, options for e-learning are still in development. Therefore, women with families and children are limited in continuing education and promotion until age 35–40 as this period is actively associated with pregnancy, parenting, and housekeeping. Likewise, sex-disaggregated data are not available [26]. Among health managers, 43% reported that the data on the personnel of their institutions, collected for Reporting to Higher Organizations, are not differentiated by gender.

### Representation of women in policy and services

The Kyrgyz Republic Country Gender Assessment ensures a representation of women and men in different managerial positions at the level of no more than 70% of employees of the same gender. This borderline is surpassed by males in executive positions in Batgen and Osh oblasts and by females in deputy positions in Bishkek and the Issyk-Kul, Naryn, and Chuy oblasts. In the Kyrgyz Republic, according to the E-Health Center data for 2019 [20], 40.5% of women hold higher executive positions in the health sector (Table 1). This percentage is higher in Bishkek and Osh (57.8% and 50%, respectively). However, in Republican institutions, only 35% of women are executive managers. Looking at the regions, there are significant diversities of levels of responsibility. The highest representation of women in the oblasts (i.e., regional districts) is in Chuy, Naryn, Talas, and Issyk-Kul oblasts (55.8, 53.1, 45.0, and 44.1%, respectively). The smallest representation of women at higher levels of responsibility is found in the southern regions of the country, with only 4.0% in the Batken oblast, 25% in the Osh oblast, and 38.7% in the Jalal-Abad oblast. The position deputy head of a healthcare institution is more frequently reserved for females. The average republic presence of women as deputy heads was 60.2%, with the highest representation in Naryn oblast, followed by Chui, and Issyk-Kul oblasts (87.5%, 72.0%, and 71.4%, respectively). The smallest percentage is found again in the Batken oblast (33.3%). In the central offices of the Ministry of Health of the Kyrgyz Republic, as of 01.01.2020, 73 people are employed, of which 46 or 63.1% are females and 27 or 36.9% are males. Looking at the oblast level, almost 40% of the heads of health organizations are women (Table 1), with women mainly represented at the primary level and men at the secondary level (hospitals) [19].

**Table 1:** The gender structure of the managerial positions in health facilities of the Kyrgyz Republic, in 2019.

Region	No of executives	No of women	%	No of Deputies	No of women	%
Kyrgyz Republic	365	148	40.5	314	189	60.2
Republic institutions*	40	14	35.0	65	30	46.2
Urban Municipality of Bishkek	45	26	57.8	66	48	72.7
Urban Municipality of Osh	8	4	50.0	8	4	50.0
Batken oblast	25	1	4.0	21	7	33.3
Jalal-Abad oblast	62	24	38.7	42	26	61.9
Issyk-Kul oblast	34	15	44.1	25	18	72.0
Naryn oblast	32	17	53.1	8	7	87.5
Osh oblast	56	14	25.0	34	19	55.9
Talas oblast	20	9	45.0	17	10	58.8
Chuy oblast	43	24	55.8	28	20	71.4

Source: E-Health Center 2020. * Republic institutions comprise specialized hospitals and research institutes at the Republic level [27].

### Gender equality in self-assessment of health managers

Based on the sample of N = 60 health managers, the results reflect the opinion of directors of health institutions, and therefore, further studies among employed health personnel are necessary to avoid the possible bias of generating the desired results based on answers which are adapted to suit national policymakers (Table 2). In addition, it is essential to include gender information in future surveys and reports which would allow for analyzing the reasons for the differences found in Table 1.

**Table 2:** Basic results of a sample of health managers in Kyrgyzstan. (Insignificant results are in italic; percent values are calculated from the sum of YES and NO answers without considering missing answers regarding a specific question (NA)).

QUESTIONS (returned questionnaires: N = 60 of 75 i.e., 80%)	YES		%	NO		%			p-value (Chi square)	NA	
**Personal Education (AE-AU included)**	**128**	**57.7**			**94**		**42.3**	-.-			**18**
AE: Is your education medical?	60	100.0			0		0.0	<0.001			0
*AM*: *Do you have a health care organizer category*?	*22*	*36*.*7*			*38*		*63*.*3*	*n*.*s*.			*0*
AO: Has the Ministry of Health of the Kyrgyz Republic evaluated your work (activity) in accordance with Order No. 724, dated June 25, 2019?	36	69.2			16		30.8	<0.01			8
AU: Are you currently taking part in the activities of the Health Management Association?	10	20.0			40		80.0	<0.001			10
**DOES YOUR ORGANIZATION HAVE THE FOLLOWING CONDITIONS?**											
**I. Computer Workplace (CR, CT, CU included)**	**146**	**98.0**			**3**		**2.0**	-.-			31
CR: Do you use a computer (software) in your work?	52	96.3			2		3.7	<0.001			6
CT: Do you consider it necessary to improve your computer skills?	52	100.0			0		0.0	<0.001			8
CU: Are you going to take courses/refresher courses in 2020–2023?	42	97.7			1		2.3	<0.001			17
**II. Training Usefulness [CD-CH included]**	**158**	**90.8**			**16**		**9.2**	-.-			66
CD: Do you think the training has helped you acquire new practical skills?	41	97.6			1		2.4	<0.001			18
CE: Do you think that the training has helped you acquire new theoretical knowledge?	45	97.8			1		2.2	<0.001			14
CF: Are you satisfied with the level (quality) of teaching this course?	39	92.9			3		7.1	<0.001			18
CH: Have you received a certificate (confirmation) of the training?	33	75.0			11		25.0	<0.01			16
**III. Digitalization [W-Z included]**	**200**	**84.0**			**38**		**16.0**	-.-			2
W: The presence of computer equipment at the manager’s workplace?	59	98.3			1		1.7	<0.001			0
X: The presence of a stable Internet connection?	56	94.9			3		5.1	<0.001			1
Y:Aavailability of technical support for computer equipment?	43	71.7			17		28.3	<0.01			0
Z: The presence of a local information network for the head (receiving electronic information from departments, including local executive bodies and accounting)	42	71.2			17		28.8	<0.01			1
**IV. Teaching Qualifications**	**54**	**37.2**			**91**		**62.8**	**-.-**			**35**
EC: Do you have experience in teaching management?	6	11.8			45		88.2	<0.001			9
ED: Are you interested in participating in management training as a teacher?	30	71.4			12		28.6	<0.01			18
EE: Does your institution have advanced (unique, specific) experience in managing an organization that is useful to show (demonstrate) to the leaders of other institutions during their training (professional development)?	18	34.6			34		65.4	<0.05			8
**V. Gender Equality (EK-EX included)**	**335**	**60.1**			**222**		**39.9**	-.-			**223**
EK: Are there problems of inequality between men and women in selection (competition) and appointment to leadership positions in health care?	11	27.5			29		72.5	<0.01			20
EL: Are there problems of inequality between men and women in earnings in similar leadership positions in health care?	32	80.0			8		20.0	<0.001			20
EM: Are there other problems of inequality between men and women in leadership positions in health care?	6	15.4			33		84.6	<0.001			21
EO: Higher authorities (MOH KR, regional coordinator, etc.) and local self-government bodies support the implementation of the Law on State Guarantees of Equal Rights and Equal Opportunities for Men and Women (Order No.97, dated July 14, 2011)	34	89.5			4		10.5	<0.001			22
*EP*: *Data on the personnel of your institution*, *collected for reports to higher organizations*, *differentiated by gender*?	*27*	*56*.*2*			*21*		*43*.*8*	*n*.*s*.			*12*
*EQ*: *Is ensuring equal rights and opportunities for men and women considered and included in the plans and programs of your institution*?	*26*	*61*.*9*			*16*		*38*.*1*	*n*.*s*.			*18*
*ER*: *Is the commitment to equal rights and opportunities for men and women reflected in the statutes and other written documents of your institution*?	*11*	*40*.*7*			*16*		*59*.*3*	*n*.*s*.			*33*
*ES*: *Over the past 5 years*, *has the number of women in leadership positions in your institution (head of a department*, *deputy director) increased*?	*29*	*60*.*4*			*19*		*39*.*6*	*n*.*s*.			*12*
ET: Does your institution’s staff have the knowledge, skills, and attitudes to do their jobs in a gender-sensitive manner?	36	73.5			13		26.5	>0.001			11
EU: Is the obligation to act in compliance with the state guarantees of equal rights and opportunities for men and women included in the job descriptions of the executives of your institution (head of a department, deputy director, director)?	27	69.2			12		30.8	<0.05			21
*EV*: *Is the obligation to act*, *observing the state guarantees of equal rights and opportunities for men and women*, *included in the job descriptions of the staff of your institution*?	*21*	*42*.*9*			*28*		*57*.*1*	*n*.*s*.			*11*
*EW*.: *Are the issues of equal rights and opportunities for men and women taken seriously by the staff of your institution*, *and are they openly discussed*?	*27*	*55*.*1*			*22*		*44*.*9*	*n*.*s*			*11*
EX: Does your institution comply with the legislation (Labor Code and other regulations) to ensure working conditions for women?	48	98.0			1		2.0	<0.001			11

*Insignificant results are in italic.

**Percent values are calculated from the sum of YES and NO answers without considering missing answers regarding a specific question (NA).

***Chi square test.

Regarding the problem levels in Gender Equality, questions EK to EX can be grouped according to the macro-level (questions EK to EO), meso-level (questions EP to ES), and micro-level (questions ET to EX). Among the directors answering the question on higher authority’s support (Table 2, question EO) 89,5% expressed the opinion that MoH, regional coordinators, and local authorities fully support the implementation of the Law on State Guarantees of Equal Rights and Equal Opportunities for Men and Women [19]. Nevertheless, a third of respondents (N = 22) considered it challenging to answer such a question. Regarding the inequalities in appointment to leadership positions in health facilities (Table 2, question EL), 80.0% of respondents experienced no inequality problems, but 20.0% did so. Correspondingly 60.4% of the respondents stated (Table 2, question ES) that in their institution the number of women in leadership positions (head of a department, deputy director) increased during the past 5 years (39.6% do not agree). This corresponds to the official data in Table 1, with an almost equal number of women in higher positions (40.5%).

Only 42.9% of the respondents reported (Table 2, question EV) that equality of rights and opportunities are integrated into the job descriptions, and an almost equal number (40.7% in Table 2, question ER), found that commitment to equal rights and opportunities for men and women is reflected in the statutory and other written documents of their institutions.

**Fig 1:**
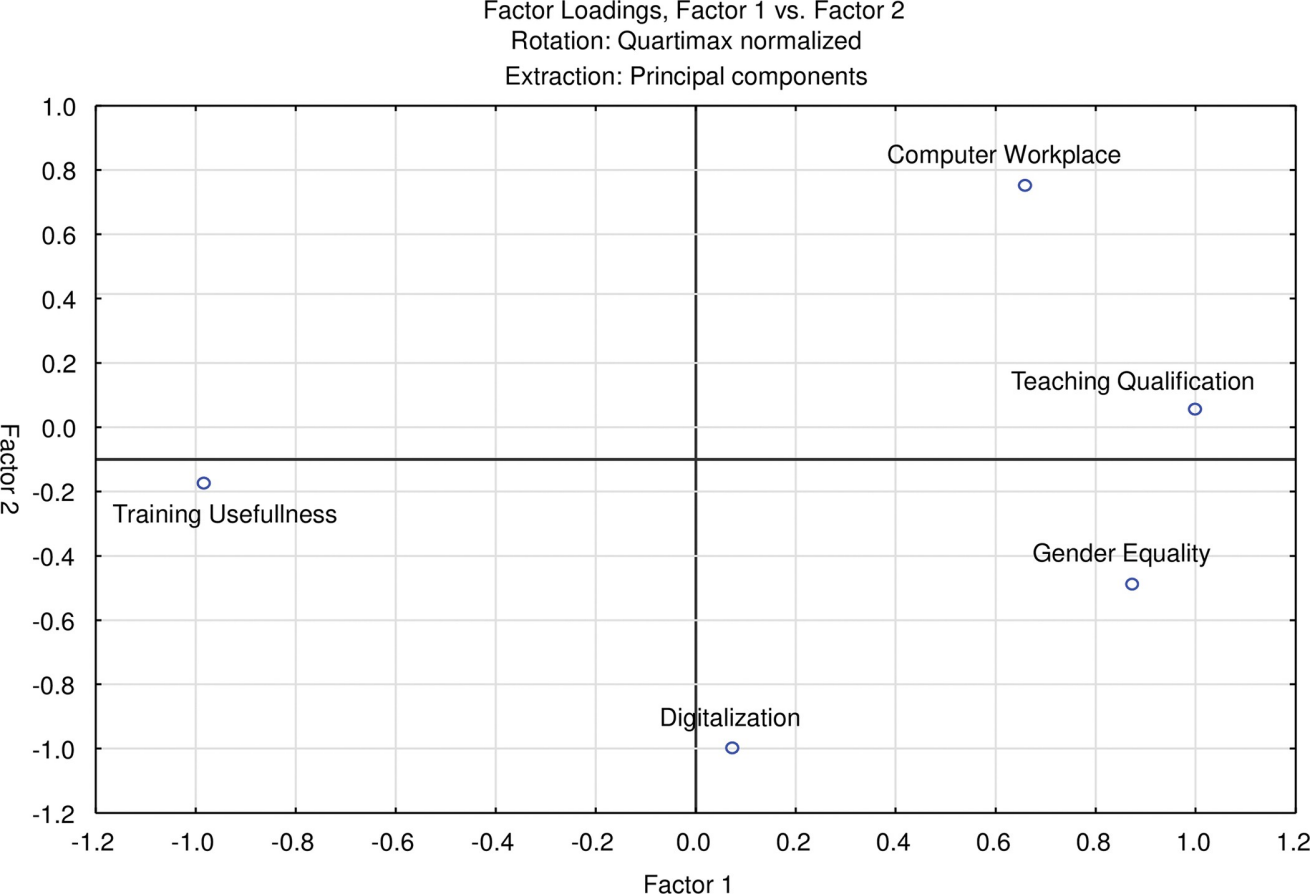
Factor Analysis with quartimax rotation of the relative positioning of Gender Equality in the management of health services in Kyrgyzstan. **Table:** 

Analyzing the variables of Table 2 –to this end grouped into five dimensions (I-V)–by a factor analysis shows a two-dimensional plot of variables that are allocated on two factors (axes). Fig 1 shows the results of the factor analysis and the factor loadings, i.e., the correlation of the variables Digitalization (W-Z), Training Usefulness (CD-CH), Computer Workplace (CR-CU), Teaching Qualification (EC-EE), and Gender Equality (EK-EX). *Factor 1 (x-axis)* could be interpreted as *personal aspects i*.*e*., the personal situation. *Factor 2 (y-axis)* relates to *technical aspects*. The factor loadings are presented as deviations from the mean normalized to zero (see red lines). High factor values mean strongly positioned above-average values compared to the other variables. A similar interpretation applies to high negative values.

**Variable**	**Factor Loadings [Quartimax normalized]** **Extraction: Principal components**	
	**Factor 1**	**Factor 2**
Gender Equality	0.859530	-0.482668
Teaching Qualification	0.981981	0.055560
Digitalisation	0.073906	-0.986580
Training Usefulness	-0.968203	-0.172708
ComputerWorkplace	0.646188	0.744582
*Expl*.*Var*	*3*.*063517*	*1*.*793625*
*Prp*.*Totl*	*0*.*612703*	*0*.*358725*

#### Relationship between Gender Equality, Training Usefulness, Teaching Qualification, Digitalization and Computer Workplace

Ordered according to their relative importance (see also the table in the legend of Fig 1), Teaching Qualification, Gender Equality, and Computer Workplace are highly correlated to personal aspects (factor 1), and Computer Workplace also to technical aspects (factor 2). Training Usefulness is highly correlated as well. It loads negatively on the first factor and is also negatively linked to the second factor, although insignificantly. However, Digitalization is negatively associated with factor two [technical aspects]. Furthermore, it is noticeable that Gender Equality, Teaching Qualification, and the Computer Workplace are building a kind of cluster of importance under personal as well as technical aspects, whereas Digitalization and Training Usefulness seem to be seen with rather mixed feelings. As mentioned above, it is impossible to indicate statistical evidence by presenting correlation coefficients between these variables because the number of cases is too small to make significant statements.

As concerns SDG 5.5 on gender equality, a detailed calculation of the two indicators 5.5.1 and 5.5.2 is placed in Annex A. These indicators measure achievements of targets described as "Ensure women’s full and effective participation and equal opportunities for leadership at all levels of decision-making in political, economic, and public life" [1]. Indicator 5.5.1 measures the proportion of seats held by women in (*a*) national parliaments (available) and (*b*) local governments (not available). Indicator 5.5.2 measures the proportion of women in managerial positions [1].

Adopting the final goal of equal participation of both genders, we calculated the years needed to achieve 50% until 2030 as the last year for the SDG achievement, based on the data for 2015 and 2020 and, alternatively, 2010 and 2020. The analysis results at 7.3 years in delay for indicator 5.2.1a (the baseline year 2015) and 15.25 year in delay (the baseline year 2010). The difference may be explained by the inherited difficult economic and political situation following the 1990ies and extending to the early 2000ds. Regarding Indicator 5.2.2, the analysis resulted in achievement of the target for 7.3 and 7.0 years before 2030, i.e., in 2023.

## Discussion

Women’s rights and gender equality in Kyrgyzstan are, to a great extent, a heritage of the Soviet period in the country’s history [28]. The historical promotion of women’s workforce participation supported women’s increased numbers and positions in education, the health sector, and other fields. However, traditional practices and patriarchal attitudes resurged after the changes of the nineties and rolled back many of the gains of the last century [29]. Challenges in achieving gender equality remain, especially regarding limited political representation and unequal pay in comparable positions [30].

However, in recent years, the Kyrgyz Republic has made serious efforts and is developing a culture of equality between women and men. The political will is essential to promote and make organizational change possible. This situation is visible in the national legislation, which supports gender mainstreaming. For example, it is recognized in the percentage of women in managerial positions (SDG 5.5, indicator 5.5.2), which increased from 32.4% in 2010 to 47.4% in 2020, which indicates that the goal of 50% equal participation can be reached by 2023 (Annex A).

Healthcare policies and programs need to take a gender-sensitive approach to ensure that they address the needs and concerns of both men and women and do not discriminate against either group [10]. All Kyrgyzstani health sector documents Manas (1996–2005), Manas Taalimi (2006–2011), Den Sooluk (2012–2018), and the current Healthy Person–Prosperous Country program 2019–2030 [20] have addressed gender equality. The Mana document of 1996 focused on improving the quality and accessibility of healthcare services in Kyrgyzstan but did not specifically address gender equality. Manas Taalimi also focused on improving the quality and accessibility of healthcare services but included a section on gender equality that outlined the importance of addressing gender-specific health needs and promoting the involvement of women in decision-making processes. The ‚Den Sooluk’ document had several specific goals and actions related to gender equality, including reducing gender disparities in access to healthcare services, improving reproductive health outcomes, and increasing the participation of women in the healthcare workforce. The most recent document, the "Healthy Person–Prosperous Country" program 2019–2030 [20], includes several goals and actions related to gender equality, including increased participation of women in the healthcare workforce, improving reproductive health outcomes, and reducing gender disparities in access to healthcare services. However, written policies at the level of healthcare facilities do not exist, and there are no documented cases of violation. Therefore, it is difficult to make a direct comparison of Kyrgyzstan’s approach to gender equality in management with that of other countries, as every country has its own unique cultural, social, and economic context [31,32]. In future surveys of health management in Kyrgyzstan, the following specific responsibilities of healthcare managers should be considered in a gender-sensitive manner at all levels [33], i.e., the macro, meso, and micro-levels:

- responsibility towards the patient, primarily within the framework of modern medicine and the movement to improve the quality of health care, within the best possible evidence and with minimal costs;
- responsibility towards employed healthcare workers, where recognition of their reasonable demands for safety in terms of personal income, adequate working conditions, and promotion, but also fears brought by the uncertainty of positive effects in their work (health outcomes of treated patients);
- the responsibility towards financiers and various interested parties who provide resources for the functioning of the institution;
- responsibility towards the community (public) in determining the way to meet the health needs of the population; and
- self-responsibility, together with the improvement of managerial knowledge and skills, and the readiness to respond effectively in conditions of constant environmental change.

Health professionals should be aware of international agreements on sexual and reproductive health and other areas of women’s health following the human rights approach embodied in these agreements. The Ministry of Health of the Kyrgyz Republic submits a report to the Ministry of Labor and Social Development two times a year. The Ministry of Labor and Social Development monitors the implementation of the action plan and holds joint discussions. However, the National Strategy of Action for Gender Equality, ending in 2020, was predominantly focused on preventing gender-based violence and equality in the family [21] and not on women’s leadership positions.

According to the stakeholder assessment (Table 2), specialists from the Ministry of Health and obstetrician-gynecologists are trained in sexual and reproductive health. They are familiar with international agreements and a human rights-based approach. There is no specific evidence about the situation in other health sectors. Regular continuing education still does not include aspects related to gender inequality and its impact on health and public health. Also, there is no evidence that evaluation for professional certification incorporates criteria related to mainstreaming a gender dimension into knowledge and practices. Gender criteria are not included in vocational assessment questionnaires. Regarding the availability of information, which highlights gender equality, the official website of the Ministry of Health and a Facebook page provides information on ongoing sector activities. However, there are no specialized sections and headings for the population.

Regarding vulnerable groups, there is assistance for victims of gender-based violence at the primary health care level, including clinical protocols, which establish the rules and conditions to ensure confidentiality and protect the rights of marginalized or stigmatized groups. Confidentiality measures are in place to protect the rights of marginalized or stigmatized groups, though evidence about their implementation is limited.

The rapid assessment pointed to still existing gender inequalities in health service, less present at the written policy level of health institutions, and more in implementation. However, the results also showed that the gender equality situation is slightly better in the health services of the Kyrgyz Republic compared to other sectors of society.

There should be a focus on fully implementing and completing the strategic and operational one-year business plan. One of the objectives of this annual business plan will concentrate on implementing written policies regarding health institutions to highlight gender equality in organizational behavior, structure, staff, and management board compositions. It will also monitor the information system in health services, which should provide gender-sensitive reporting (actually not always the case, as seen from this assessment).

With the support of the Government of Switzerland, within the "Health Facility Autonomy project, Phase II (HFA Phase II), a specific module on human rights and gender equality in health care services will be developed and become the composite part of the training strategy: "The human rights and gender equality in health care services" based on the WHO approach [3] and its guidelines leading to gender mainstreaming for health managers. Such a module will train management staff in gender analysis for institutional planning, organizing, staffing, leading, and evaluating controls. The module will provide the Kyrgyz health managers with competencies to deal with health workers to be aware of and exercise their human and gender rights.

Nevertheless, making health organizations gender-responsive needs more time, despite health workers dealing with gender in everyday practice as one of the most critical social determinants of health and diseases [34].

## Conclusions and recommendations

At the national health policy level, the Kyrgyz Republic has developed policies and initiatives that support the advancement of women in the workforce and promote gender equity in all areas, including in management and leadership positions. Overall, the Kyrgyzstan health sector documents have become increasingly focused on addressing gender equality over time. Gap analysis pointed to this trend regarding SDGs related to gender issues. The self-assessment revealed that women are less present in the top managerial positions. Also, Gender Equality, Teaching Qualification, and the Computer Workplace are building the cluster of importance under personal as well as technical aspects. Despite this improvement, the gender equality approach needs better integration at the policy level of institutions and implementation in the Kyrgyz Republic. Therefore, the following is proposed:

- to develop a written policy that affirms a commitment to gender equality in organizational behavior, structures, and staff and management board compositions, together with preparing strategic and operational plans of health care institutions;
- to train management staff in gender analysis for institutional planning, organizing, staffing, leading, and evaluating/controlling, but also health workers to be aware and exercise their human and gender rights;
- to integrate gender sensitivity into human resource development for staff at all levels to improve organizational effectiveness, promote non-discriminatory relationships, and respect for diversity in work and management styles;
- to include gender awareness in job performance criteria with striving to increase the number of women in senior decision-making positions and on boards of directors;
- to institute family-friendly policies and create an environment that enables both women and men to balance work and family life together with that, which supports equal pay for equal work;
- to establish a mechanism consistent with the organization’s mission and objectives, which allows proper sex disaggregation of institutional data according to gender approach.

## Supporting information

S1 Appendix Appendix A: SDG 5.5. (DOCX)

S2 Appendix Appendix B: Consent form. (DOCX)

S3 Appendix Appendix C: PLOS ONE KYRG EXCEL II indiv. answers FULL 230113. (XLSX)
