# Supplementary material for: Rights and responsibilities: Women leadership for health in Kyrgyzstan
Source: PLoS One. 2024 Feb 16;19(2):e0295239. doi: 10.1371/journal.pone.0295239 (PMC10871472; doi:10.1371/journal.pone.0295239)
Supplement: S1 Appendix — (DOCX) [file pone.0295239.s001.docx]

**Supporting Information**

**Appendix A: SDG 5.5**

Note: The calculations are done following the scheme provided by Bjegovic-Mikanovic et al. in:

[35] Bjegovic-Mikanovic V, Salem ZA, Wenzel H, Broniatowski R, Nelson C, Vukovic D, Laaser U. A gap analysis of SDG 3 and MDG 4/5 mortality health targets in the six Arabic countries of North Africa: Egypt, Libya, Tunisia, Algeria, Morocco, and Mauritania. Libyan Journal of Medicine 2019: 14/1. Available at: <https://doi.org/10.1080/19932820.2019.1607698>

**Quantifiable SDG-5.5 targets and indicators**

**SDG 5.5**

Ensure women's full and effective participation and equal opportunities for leadership at all levels of decision-making in political, economic, and public life.

#### Target 5.c Adopt and strengthen sound policies and enforceable legislation for the promotion of gender equality and the empowerment of all women and girls at all levels

**Indicators available for target 5.c^[[1]](#footnote-1)^**

##### Indicator 5.5.1: Proportion of seats held by women in [a] national parliaments [available] and [b] local governments [not available].

Indicator 5.5.1.a: Proportion of women in national parliaments.

2010: 25.6%

2015: 23.3%

2020: 19.2%

Indicator 5.5.2: Proportion of women in managerial positions

2010: 32.4%

2015: 35.7%

2020: 47.4%

The likelihood of achieving the SDG targets in 2030 will be determined by the indicators' time gaps, i.e. the time remaining to achieve an agreed target. To this end, we use the mathematical model of the United Nations Development Program [UNDP] used to assess progress toward the SDGs according to the following equations:

Tr - remaining time

Tn – time needed to achieve the target [in linear progress]

G – time gap [gain or delay]: Tr – Tn

Gr = G / Tr >= −0.25

Tt – target year

Tc _–_ year of observation

Tb – baseline year

Xb – baseline value of the indicator

Xt – target value of the indicator

Xc – observed value of the indicator Tr = tt − tc

and the formula: Tn = tt − [tb + [tt − tb] *X c* − *Xb / X t* − *Xb*]

Then, the resulting time-gap G is calculated as: G=Tr−Tn

A positive time-gap G indicates that the respective country is 'On Track' to achieve the target on time or even earlier; a negative value indicates that it may still be 'Likely' or even 'Unlikely' to achieve the target within the target timeframe, i.e. in 2030. A country is still considered likely to achieve the target as long as a negative value for G does not make up for less than −25% of the remaining time T_r_ i.e. the relative Gap G_r_ is: Gr = G / Tr >= −0.25

**Then Kyrgyzstan's chances to achieve SDG 5.5 - set as women's share of 50% - in time are as follows:**

**A] Indicator 5.2.1: the observed year 2020 and baseline year 2015**

Tr = 2030 – 2020 = 10

Tn1 = tt − [tb+[tt−tb] Xc−Xb/Xt−Xb]

2030 – [2015+[2030-2015]19.2-23.3/50-23.3]

2030 – [2015 + 15 x -4.1/26.7

2030 – [2015 + 15 x -0.1536

2030 – [2015 - 2.3]

2030 – 2012.7

**Tn1 = 17.3**

**G = 10 – 17.3 = -7.3**

**Gr = -7.3 / 10 = -0.73 > -0.25**

**B] Indicator 5.2.1: observed year 2020 and baseline year 2010**

Tn2 = tt−[tb+[tt−tb] *Xc*−*Xb/Xt*−*Xb*]

2030 - [2010 + [2030 - 2010] 19.2-25.6/50-25.6]

2030 – [2010 +[ 20] x -6.4/24.4]

2030 – [2010 + 20 x -0.2623]

2030 – [2010 - 5.25]

2030 – 2004.75

**Tn2 = 25.25**

**G = 10 – 25.25 = -15.25**

**Gr = -15.25 / 10 = -1.53 < -0.25**

**C]** **Indicator 5.2.2: observed year 2020 and baseline year 2015**

Tr = 2030–2020 = 10

Tn3 = tt−[tb+[tt−tb] *Xc*−*Xb / Xt*−*Xb*]

2030 – [2015+[2030-2015] 47.4-35.7/50-35.7]

2030 – [2015 + 15 x 11.7/14.3]

2030 – [2015 + 15 x 0.8182]

2030 – [2015 + 12.3]

2030 – [2027.3]

**Tn3 = 2.7**

**G = 10 – 2.7 = 7.3**

**Gr = 7.3 / 10 = 0.73 > -0.25**

**D] Indicator 5.2.2: observed year 2020 and baseline year 2010**

Tn4 Tn=tt−[tb+[tt−tb] *Xc*−*Xb/Xt*−*Xb*]

2030 – [2010+[20] 47.4-32.4/50-32.4]

2030 – [2010 + 20 x 15/17.6]

2030 – [2010 + 17.0]

2030 – [2027]

**Tn4 = 3**

**G = 10 – 3 = 7**

**Gr = 7 / 10 = 0.7 > -0.25**

1. At: <https://unstats.un.org/sdgs/dataportal/analytics/DataAvailability> [↑](#footnote-ref-1)
