# Supplementary material for: Rights and responsibilities: Women leadership for health in Kyrgyzstan
Source: PLoS One. 2024 Feb 16;19(2):e0295239. doi: 10.1371/journal.pone.0295239 (PMC10871472; doi:10.1371/journal.pone.0295239)
Supplement: S2 Appendix — (DOCX) [file pone.0295239.s002.docx]

**Appendix B: Consent form**

**INFORMED CONSENT FORM FOR PARTICIPANTS OF STUDY:**

**Gender and inclusion analysis in health service in the Kyrgyz Republic**

**We invite you to participate in this study of gender inequalities at all levels in the Kyrgyz Republic health sector, which can influence appropriate, effective, efficient health care management, quality and service delivery.**

Before deciding whether to participate, it is important that you understand why this study is being done and what it entails. Please read the following information carefully and, if you wish, discuss it with people you would like to consult if you feel it is necessary. If something is unclear or if you would like more information, we will be happy to answer your questions. You have plenty of time to decide if you agree to participate.

Thank you for taking the time to read this information.

1. **The name of the study and keywords**

The name of this study is: Gender and inclusion analysis in health service in the Kyrgyz Republic and EuroHealth is performing it within the frame of the HFA (Health Facility Autonomy) project, with the basic aim to provide necessary information for strengthening health facility management. Keywords: health management, health facility, gender equality, gender inclusion

1. **Explanation/ Background**

Today many countries take inspiration from the UN agenda for sustainable development. Within SDG 5 dedicated to gender equality, SDG 3 dedicated to health/ wellbeing and all gender-related SDGs, countries are trying to empower women and promote gender equality in governance and management at the macro (society and policy), meso (communities and institutions) and micro (social interactions in departments) levels. WHO approach to gender mainstreaming also refers both to projects and institutions striving to build capacities in developing gender equality, promoting the use of sex-disaggregated data and gender analysis, and establishing accountability. Since women account on average for 70% of the workforce for health, gaps in health workers will decrease only by addressing the gender dynamics of the workforce. Gender parity in health management differs from country to country, but always men prevail at the top positions of health care organizations. Similar disparities between women and men are present during reimbursement for their work; usually women are underpaid. These gaps in many countries are more present in the health sector than in other areas, which is largely unexplained.

The legislation of the Kyrgyz Republic in the field of gender relations is based on the Constitution of the Kyrgyz Republic, international treaties and agreements to which the Kyrgyz Republic expressed full commitment. The Law on State Guarantees of Equal Rights and Equal Opportunities for Men and Women and other legal acts of the Kyrgyz Republic support the gender equality. One of the most important is the Program of the Kyrgyz Republic Government on Public Health Protection and Health Care System Development for 2019-2030, which is opening solid base for gender mainstreaming in the health sector. According to the national legislation, persons of different sexes compete on equal terms and have equal pay with the same qualifications of the employee and the same working conditions.

**3. Objectives of the Study**

The main objective of the study is to assess possible gender inequalities at all levels in the Kyrgyz Republic health sector, which can influence appropriate, effective, efficient health care management, quality and service delivery.

## CONSENT FOR PARTICIPATION IN STUDY:

**Gender and inclusion analysis in health service in the Kyrgyz Republic**

**Full name of the participant: ____________________________________________________________**Participant number: _______

With signing of this document, I do confirm the following:

- I have had enough time to consider my involvement and have had the opportunity to ask questions and have received satisfactory answers to all my questions.
- I do understand that my participation is voluntary.
- I understand that solely researchers can have access to my questionnaire to ensure that the data is properly recorded. All personal information will be considered as STRICTLY CONFIDENTAL.
- I understand that the data collected during my participation in this assessment of gender equality is entered into a database and analyzed and will only be used for scientific purposes.

**If you agree to participate in this study of assessment of gender equality in the management of my health organization**

**click *HERE***
